# Prognostic Value and Therapeutic Perspectives of CXCR Members in the Glioma Microenvironment

**DOI:** 10.3389/fgene.2022.787141

**Published:** 2022-04-27

**Authors:** Jiarong He, Zhongzhong Jiang, Jiawei Lei, Wen Zhou, Yan Cui, Biao Luo, Mingming Zhang

**Affiliations:** ^1^ Department of Neurosurgery, The Second Xiangya Hospital, Central South University, Changsha, China; ^2^ Department of General Surgery, The Second Xiangya Hospital, Central South University, Changsha, China

**Keywords:** CXCR, tumor microenvironment, glioma, mutation burden, prognosis

## Abstract

**Background:** CXCR (CXC Chemokine Receptor) is a complex of the immune-associated protein involved in tumor activation, invasion, migration, and angiogenesis through various chemical signals in the tumor microenvironment (TME). However, significant prognostic characteristics of CXCR members and their impact on the occurrence and progression of glioma have not yet been fully elucidated.

**Methods:** In this research, we used Oncomine, TCGA, GTEx, and CGGA databases to analyze the transcription and survival data of glioma patients. Afterward, the influence of CXCR members on the TME was explored using comprehensive bioinformatics analysis.

**Results:** The mRNA expression levels of CXCR1/2/3/4/7 were significantly up-regulated in glioma than in normal samples, whereas the mRNA expression level of CXCR5 was decreased. We then summarized the genetic alteration landscape of CXCR and identified two molecular subtypes based on CXCR expression patterns in glioma. The characteristics of CXCRs were also investigated, including the clinicopathological parameters, TME cell infiltration, and prognosis of patients with glioma. After Lasso and multivariable Cox regression, a CR-Score for predicting overall survival (OS) was constructed and the predictive performance of the signature was validated. The high-risk group was a significantly poorer prognostic group than the low-risk group as judged by the CR-Score (TCGA cohort, *p* < 0.001, CGGA cohort, *p* < 0.001). Moreover, the CR-Score was significantly correlated to the tumor-immune infiltration and cancer stem cell (CSC) index. A risk scale-based nomogram incorporating clinical factors for individual risk estimation was established thereby.

**Conclusion:** These findings may pave the way for enhancing our understanding of CXCR modification patterns and developing better immune therapeutic approaches for glioma.

## Introduction

Despite recent advances in therapeutic options such as surgery, radiotherapy, and chemotherapy, the prognosis of glioma patients remains unsatisfactory (Sung et al., 2021). The introduction of immune checkpoint inhibitors (ICIs) has become a breakthrough in tumor immunotherapy in recent years. Unfortunately, only a minority of glioma patients respond to ICIs (Romani et al., 2018), and the incidence of anti-PD-L1 treatment-related adverse events is up to 16% (Marin-Acevedo et al., 2019). Therefore, there is a need for the development of more effective regimens that are better tolerated and more efficient. The efficacy of checkpoint blockade immunotherapy largely depends on the composition and proportion of tumor-infiltrating lymphocytes (TILs) (Curran et al., 2010). Tumors with high TIL content are called immunologically active “inflamed” tumors and usually respond to ICI (Pitt et al., 2016; Cristescu et al., 2018), while immunologically inactive “ non-inflamed” TIL (-) tumors do not benefit from immune checkpoint blockade. The interaction of tumor cells with the tumor microenvironment plays a critical role in cancer progression, aggressiveness, and response to immunotherapy (Wang et al., 2017).

In the last decades, the chemokine system has been widely studied in multiple cancer cell lines (Mollica Poeta et al., 2019). CXC chemokine receptors, a diverse group of 7-transmembrane domains G protein-coupled receptor, are frequently involved in tumorigenesis and tumor progression (Mollica Poeta et al., 2019). CXCR1 and CXCR2 are cellular membrane receptors for Interleukin-8 receptor A (IL-8RA) and Interleukin-8 receptor B (IL-8RB), respectively, mainly expressed on the surface of T cells, monocytes, and neutrophils, belonging to the GPCR superfamily (Jin et al., 2019). CXCL8/CXCR1 performs as drug receptors and signal transduction, while CXCL8/CXCR2 promotes inflammation and angiogenesis (Park et al., 2012). Overexpression of CXCR1 and CXCR2 strengthened the invasion capability of tumor cells. Jin et al. reported that CXCR1 and CXCR2 modified CARs significantly enhanced the persistence and migration of T cells in tumors, inducing tumor degeneration and persistent immunologic memory in preclinical models of malignancies such as glioblastoma, pancreatic and ovarian cancer (Jin et al., 2019).

CXCR3 is a crucial molecule in tumorigenesis and neuroinflammatory. Zhou Y et al. discovered that CXCR3 was also involved in the pathogenesis of glioma, chronic pain, bipolar disorder, MS, AD, and HAM/TSP (Zhou et al., 2019). Previous studies have found that the dysregulation of CXCR3 was negatively correlated with tumor invasion depth (Hu et al., 2015). Meanwhile, it regulates the activation of TILs and resident immune cells (Zhou et al., 2019). CXCR4 as the most common type of GPCR member stands out for its involvement in several pathological conditions, including immune diseases and cancer (Pozzobon et al., 2016). The expression levers of the CXCR4 and its ligand stromal cell-derived factor-1(SDF-1, CXCL12) are maintained by chemokine signaling pathways via positive feedback loops. Recently, the expression of CXCR4 was found to be involved in cancer stem cells self-renewal and the generation and maintenance of the perivascular stem cell niche (Richardson, 2016). Moreover, extracellular regulated kinase (ERK) pathway, transforming growth factor (TGF)-alpha and matrix metalloproteinase (MMP)-7, MMP-9 were found closely associated with the expression of CXCR4 (Fanelli et al., 2012). CXCR5 was closely related to tumor progression. Yang et al. discovered that glioblastoma cells target CXCR5 by releasing exosome miR-214-5p to regulate lipopolysaccharide stimulation to modulate microglia inflammatory response (Yang et al., 2019). CXCL16/CXCR6 axis acts a pivotal part in the pro-tumor microenvironment, and the silencing of CXCR6 reduced the proliferation rate on glioma cells (Lepore et al., 2018), indicating that CXCR6 plays an oncogenic role in glioma. In addition, CXCR7, also known as ACKR3, a new functional receptor for CXCL12 with a higher affinity than CXCR4, mediates resistance to drug-induced apoptosis. Previous studies have also shown that CXCR7 is significantly associated with adverse outcomes (Hattermann et al., 2010).

To date, the dysregulated expression of CXCR members and their significant prognostic role have been partly studied in some researches. Most of the previous studies evaluate the performance of one or two CXCRs due to technical limitations, while the immune response is characterized by multiple genes interacting in a highly coordinated manner. Thus, a comprehensive analysis of the characteristics of multiple CXCR-mediated cell infiltrates may provide additional insights into the prediction of immunotherapy responses and the underlying mechanisms of glioma tumorigenesis. In this report, we identified the expression and potential prognostic value of CXCRs for glioma patients through computational analysis. The genome information from 1,360 glioma samples was incorporated to correlate the chemokine system with the immunity characteristics of the tumor-associated microenvironment. Our study concludes that the CR-Score is a reliable prognostic predictive value for glioma and can inform special immunotherapy treatment.

## Materials and Methods

### Data Collection and Preprocessing

The workflow diagram of this research was shown in Supplementary Figure S1. Gene expression data for normal brain tissues were obtained from the Genotype-Tissue Expression (GTEx) Data Portal (https://xenabrowser.net/datapages/). Original RNA-sequencing (RNA-seq) data (fragments per kilobase per million fragments mapped, FPKM) and corresponding clinicopathological features of 1,360 glioma patients were downloaded from The Cancer Genome Atlas (TCGA) database (http://Cancegenome.nih.gov/) and Chinese Glioma Genome Atlas (CGGA) platform (http://www.cgga.org.cn/). The detailed information on the samples is presented in Table 1. Patients without survival information were excluded from the corresponding analysis. The FPKM values were converted into transcripts per kilobase per million (TPM) before further investigation (Conesa et al., 2016). All the datasets were retrieved from the published literature and the ethics statement confirmed that all written informed consent was obtained.

**TABLE 1:** Clinical characteristics of patients with glioma.

Characteristics	CGGA Cohort (n=693)	TCGA Cohort (n=667)
**Age**		
≤65	661 (95.4)	535 (80.2)
>65	31 (4.5)	132 (19.8)
NA	1 (0.1)	0
**Gender**		
Male	398 (57.4)	390 (58.5)
Female	295 (42.6)	277 (41.5)
**Histologic grade**		
2	188 (27.2)	149 (22.4)
3	255 (36.8)	159 (23.8)
4	249 (35.9)	359 (53.8)
NA	1 (0.1)	0
**Survival status**		
OS years (median)	3.28	1.95
Alive	266 (38.4)	371 (55.6)
Dead	397 (57.3)	296 (44.4)
NA	30 (4.3)	0
**PRS-type**		
Primary	422 (60.9)	647 (97.1)
Recurrent	271 (39.1)	20 (2.9)
**Radio-status**		
treated	510 (73.6)	493 (73.9)
un-treated	136 (19.6)	151 (22.7)
NA	47 (6.8)	23 (3.4)
**Chemo_status**		
TMZ-treated	486 (70.2)	-
un-treated	161 (23.2)	-
NA	46 (6.6)	-

### Consensus Clustering of Differentially Expressed Genes

In this study, we used the “edger” R package to normalize the transcriptome sequencing data of the two cohorts before comparison. Limma package was utilized for differential expression analysis with |log2FC| ≥1 and FDR <0.05. *p* values were denoted as follows: *, *p* < 0.05; **, *p* < 0.01; ***, *p* < 0.001. Clustered analysis of CXCR-mediated patterns was employed using the Genesis K-means method. The “ConsensuClusterPlus” package was used to control the stability and optimal of clusters. In addition, the differences in overall survival rates between the two subtypes were evaluated by the Log-rank test using the survminer R package.

### Association of Two Clusters With Tumor Microenvironment and Immune Checkpoint Blockade in Glioma

ESTIMATE algorithm was utilized to evaluate the stromal/immune scores of each glioma sample. Furthermore, we used the CIBERSORT algorithm to calculate the abundance of 22 immune cell subsets for each tumor specimen (Newman et al., 2015). Also, the expression of immune checkpoint blockade between the two clusters was analyzed.

### Analysis of Mutation and Copy Number Variation

We used the R package “maftools” to generate waterfall maps of genomic mutations and copy number variations (CNVs) in the TCGA-glioma cohort (VarScan2). For copy number variation analysis, GISTIC.2 was used to identify missing gene sequences and amplified genomes, including deep deletion, shallow deletion, high amplification, and low-level gain. The gain or loss in copy number was determined by the total number of genes with copy number alterations at the focal and arm levels. The tumor mutation burden (TMB) was calculated as the number of all somatic copy number alterations (SCNAs) using the two-sample t-test.

### Development and Validation of the Prognostic Model

Univariate and multivariate Cox proportional hazard analyses were performed to assess the prognostic factors, including CR-Score, patient age, gender, and tumor grade. Hazard ratios (HR) and corresponding 95% confidence intervals (CI) were estimated using the R package “forestplot”. The R package “glmnet” was used for Lasso-penalized Cox regression analysis to construct a prognostic model. We set the significance cut-off *p*-value as 0.05, and five CXCRs were selected and used for further analysis.

The CR-Score was calculated using the following equation:

$$\text{CR}‐\text{Score}=\sum \left(\text{Exp}{\left(\text{Xi}\right)}^{*}Coef\left(Xi\right)\right)$$

where Exp (Xi) and Coef (Xi) represented the expression and coefficient of each gene Xi, respectively. Principal component analysis (PCA) was conducted in R using the “prcomp” function. The predictive capability of the CR-Score was evaluated using Receiver Operating Characteristic (ROC) curve analysis.

### Functional Annotation and Immune Infiltration Analysis

For gene ontology (GO) pathway and Kyoto Encyclopedia of Genes and Genomes (KEGG) analysis, we used the clusterProfiler R package. In addition, we performed the Single-sample gene set enrichment analysis (ssGSEA) to quantify the relative abundance of immune infiltration levels using the “GSVA” R package (Rooney et al., 2015). The Timer, Quantiseq, Mcpcounter, Xcell, and Epic databases were used to calculate the fractions of infiltrating immune cells in glioma.

### Establish a Predictive Nomogram Scoring Model

Based on the results of the independent prognostic analysis, the “rms” R package was used to construct a nomogram to predict personalized survival probability. Each clinicopathologic variable was assigned an integer-weighted score in the nomogram scoring model. The sum of all variables scores was added up to get the total score. Clinical ROC was performed to assess the predictive efficiency of the nomogram model. The calibration plot was used to evaluate the accuracy of the prediction for the probability of survival events at 1-, 3-, and 5- year.

### Statistical Analysis

Pearson and Spearman correlation coefficients were used to determine the correlation between two variables. We used the unpaired student’s t-test to assess the statistical significance for normally distributed variables, and the Mann-Whitney U test was used to analyze non-normally distributed continuous variables. A one-way ANOVA test was used to compare two or more groups. Survival curves of the two subgroups in each data set were estimated by Kaplan-Meier method and compared by the log-rank (Mantel-Cox) test. All statistical analyses were accomplished with R software (v4.0.2, https://www.r-project.org/), with a *p*-value < 0.05 (two-tailed test) indicating statistical significance.

## Results

### Identification of Transcriptional Variations and Genetic Alterations of CXCRs in Glioma

**FIGURE 1:**
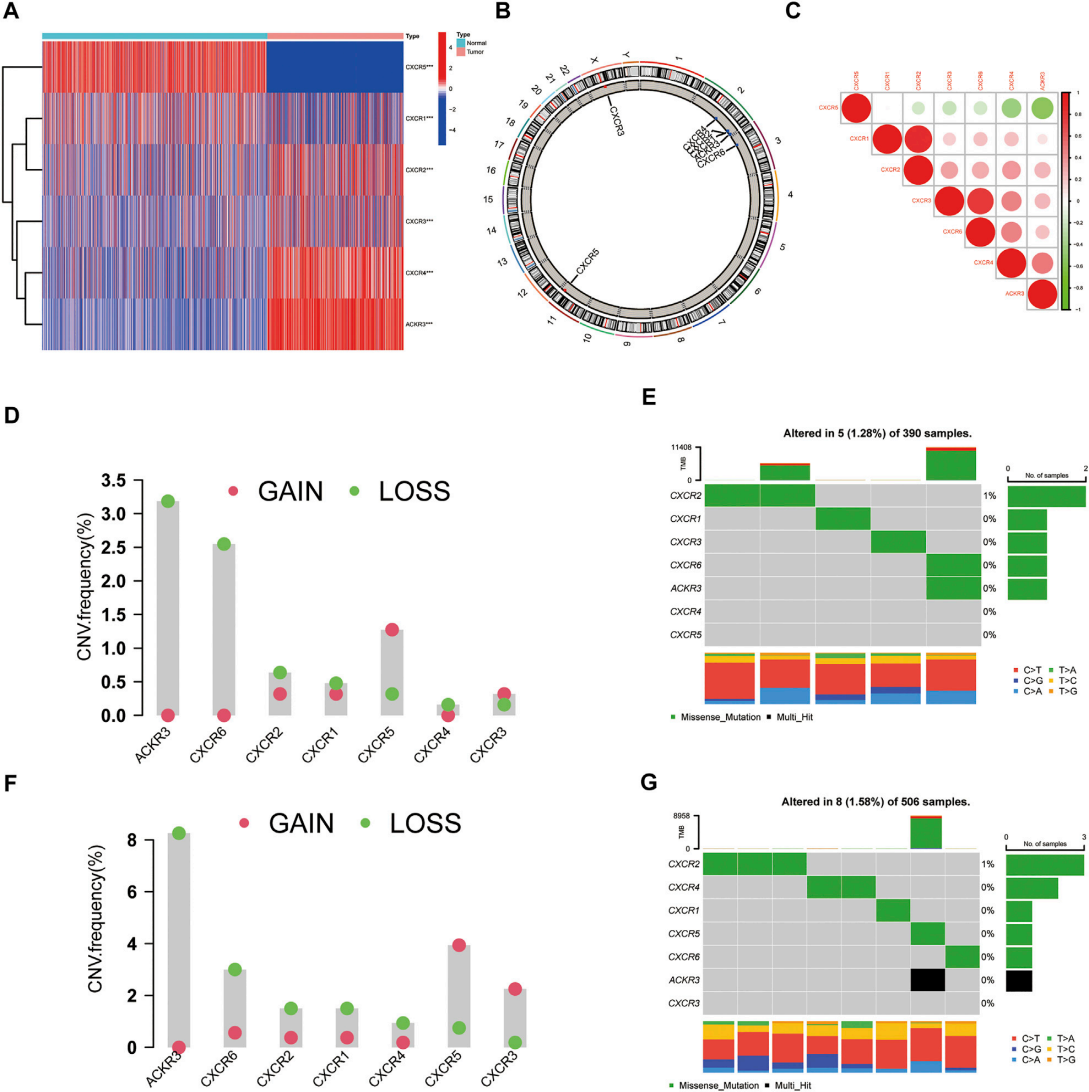
Transcriptional and Genetic alterations of CXCRs in glioma. **(A)** Heatmap of six differential expression CXCRs in glioma tissues and normal brain tissues (red: high expression; blue: low expression). Asterisk indicates: t test, *p < 0.05, **p < 0.01 and ***p < 0.001 compared to the normal brain tissues. **(B)** The location of CNV alteration of seven CXCR molecules on 23 chromosomes. **(C)** Correlations between seven CXCR genes. The darker the blue, the stronger the negative correlation, and the darker the red, the stronger the positive correlation. CNV distribution of CXCRs in GBM **(D)** and LGG **(F)** tumor samples. The height of the bar represented the total alteration frequency. The landscape of somatic mutation frequency of CXCRs in GBM cohort **(E)** and LGG cohort **(G)**. The somatic mutation landscape of the GBM cohort **(E)** and LGG cohort **(G)**. Each column represented an individual sample. The bar graph on the right indicated the mutation frequency of each gene. The upper barplot showed the mutations.

Gene expression levels of seven CXCRs were measured between 667 tumor and 1,431 normal brain tissues from TCGA-Glioma and GTEx-Brain data. A total of six CXCRs were either downregulated or upregulated in glioma (Figure 1A). Among them, one gene (CXCR5) was downregulated while five other genes (CXCR1, CXCR2, CXCR3, CXCR4, and ACKR3) were enriched in glioma compared with normal brain tissues (Supplementary Table S1). Oncomine platform was used to compare the mRNA expression levels of CXCRs in pan-cancer tissues and normal tissues (Supplementary Figure S2).

Furthermore, we identified the distributions of copy number variation in the chromosomes (Figure 1B). The correlation network analysis of seven CXCR molecules was presented in Figure 1C. We also investigated the frequency of CNV alterations and found that more than half of the seven CXCRs had copy number deletions (Figures 1D–F). At the genetic level, five of 390 (1.28%) glioblastoma (GBM) samples and eight of 506 (1.58%) low-grade glioma (LGG) samples confirmed genetic mutations (Figures 1E–G). Figure 1D demonstrated that ACKR3 with the highest frequency of variants in GBM, followed by CXCR6. Compared to the LGG cohort, ACKR3 also showed the highest mutation frequency, among the seven CXCRs. We further found that the mRNA expression of ACKR3 was up-regulated, showing CNV loss, while the down-regulation of CXCR5 showed CNV gain, indicating that CNV alteration might regulate the transcriptional activity of CXCRs.

### The Characteristics of CXC Chemokine Receptor Subtypes in Glioma

**FIGURE 2:**
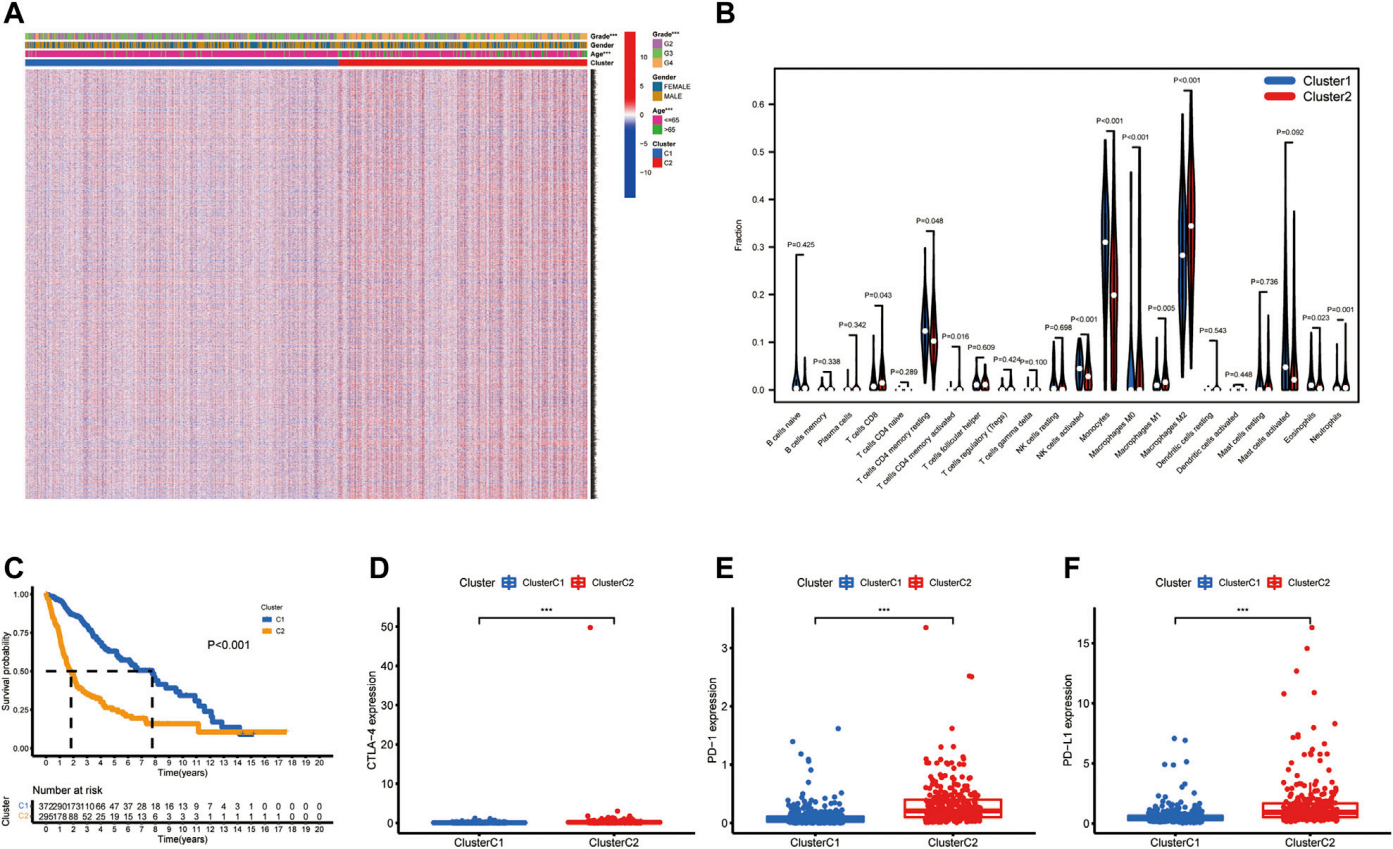
The clinicopathological and TME characteristics of the two molecular subtypes. **(A)** Heatmap showing clinicopathologic features of the two clusters. **(B)** The proportions of 22 immune cells among the clusters. **(C)** Kaplan–Meier curves for OS of the two clusters (log-rank tests, *p* < 0.001). **(D–F)** Histograms representing CTLA-4, PD-1, and PD-L1 expression. Data are shown as the mean ± SD; t test, *p < 0.05, **p < 0.01 and ***p < 0.001 compared to the cluster C2 in **(D–F)**.

To investigate the relationship between the expression of these seven CXCR genes and glioma subtypes, we performed a consensus clustering analysis in glioma patients. By applying the standard K-means clustering algorithm, when k = 2, the inter-group correlation is low and the intra-group correlation is the highest. The results showed that 667 glioma patients were divided into two separate clusters (C1, *n* = 372; C2, *n* = 295). Gene expression profiles (GEPs) and corresponding clinicopathological parameters including gender (male or female), age (≤65 or >65 years), and tumor histological differentiation (G2-G4) were presented in a heatmap (Figure 2A). To examine the effect of CXCRs on the TME of glioma, we used the CIBERSORT algorithm to assess the diversities between the two subtypes from cell level (Supplementary Table S2). Among them, the infiltration levels of T cells CD4 memory resting, NK cells activated, Monocytes, and Eosinophils were significantly higher in cluster C1 than in cluster C2 (Figure 2B). Moreover, a significant difference for OS was found in the two clusters (Figure 2C). The results showed that the expression levels of immune checkpoints in cluster C2 were significantly higher than those in cluster C1 (Figures 2D–F).

### Construction and Evaluation of a Prognostic Model

**FIGURE 3:**
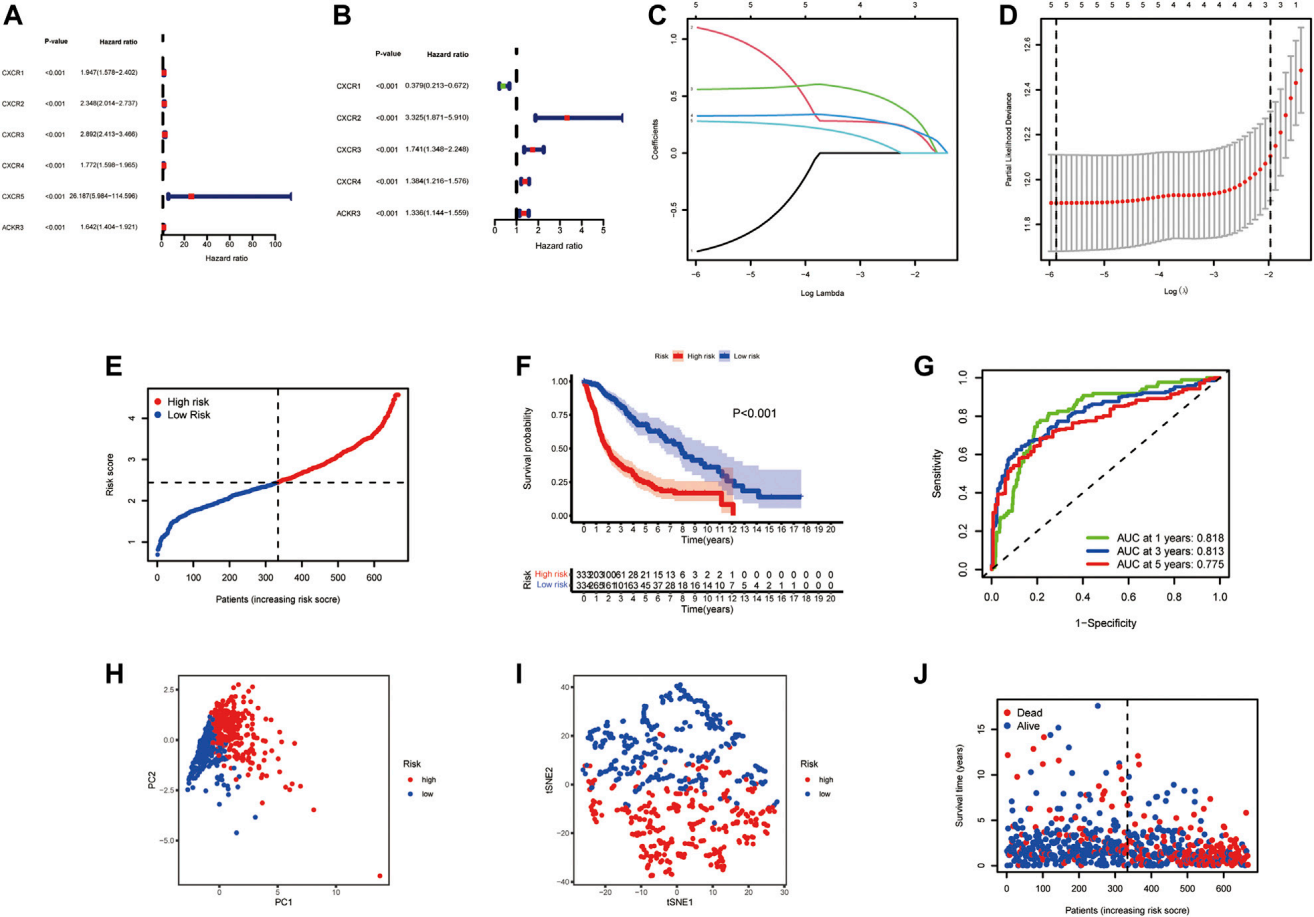
Prognostic value of the risk signature model. Forest plot of prognostic CXCR genes based on univariate **(A)** and multivariable **(B)** Cox regression analysis. **(C)** Lasso coefficient profiles of the five survival-related CXCRs. **(D)** Ten‐fold cross‐validation for tuning parameter selection in the Lasso model. **(E)** The distribution of CR-Score in the TCGA dataset. **(F)** Kaplan‐Meier curve of OS in the high- (red) and low-(blue) risk groups. **(G)** ROC curves for the predictive model. **(H)** PCA plot of the TCGA cohort. **(I)** t-SNE plot for patients. **(J)** Distribution of the survival data and CR-Score.

To construct a CXCR-related prognostic model, univariate and multivariable Cox regression analyses were used to screen for survival-related genes. Univariate Cox regression analysis revealed the prognostic value of the six CXCRs in glioma patients (Figure 3A and Supplementary Table S3). Subsequently, five genes with independent prognostic value (CXCR1, CXCR2, CXCR3, CXCR4, and ACKR3) were identified by multivariate Cox regression, of which 4 genes (CXCR2, CXCR3, CXCR4, and ACKR3) had an increased probability of death (HR > 1), while the remaining CXCR1 gene was a protective factor for HR < 1 (Figure 3B and Supplementary Table S3). The 5-gene signature was identified by Lasso penalized Cox regression analysis based on the optimum λ value (Figures 3C,D). The CR-Score of the CXCR-based model was calculated as follows: CR-Score = (−0.9711 * CASP1 exp.) + (1.2015 * CXCR2 exp.) + (0.5543 * CXCR3 exp.) + (0.3252 * CXCR4 exp.) + (0.2893 * ACKR3 exp.). Patients were divided into the high-risk group (*n* = 333) and the low-risk group (*n* = 334) according to the median cut-off value (Figure 3E). Kaplan-Meier survival analysis of glioma patients showed that high CR-Scores were associated with significantly worse patient survival (Figure 3F, *p* < 0.001). In addition, the effectiveness of the model was assessed by time-correlated ROC analysis, and the area under the curve (AUC) for the signature was 0.818 at 1 year, 0.813 at 3 years, and 0.775 at 5 years for survival (Figure 3G). Through principal component analysis and t-distributed Stochastic Neighbor Embedding (t-SNE), it was found that patients with different CR-Scores had different directions of distribution (Figures 3H,I), and patients in the low-risk group had better OS than those in the high-risk group (Figure 3J).

### Validation of the Prognostic Model

**FIGURE 4:**
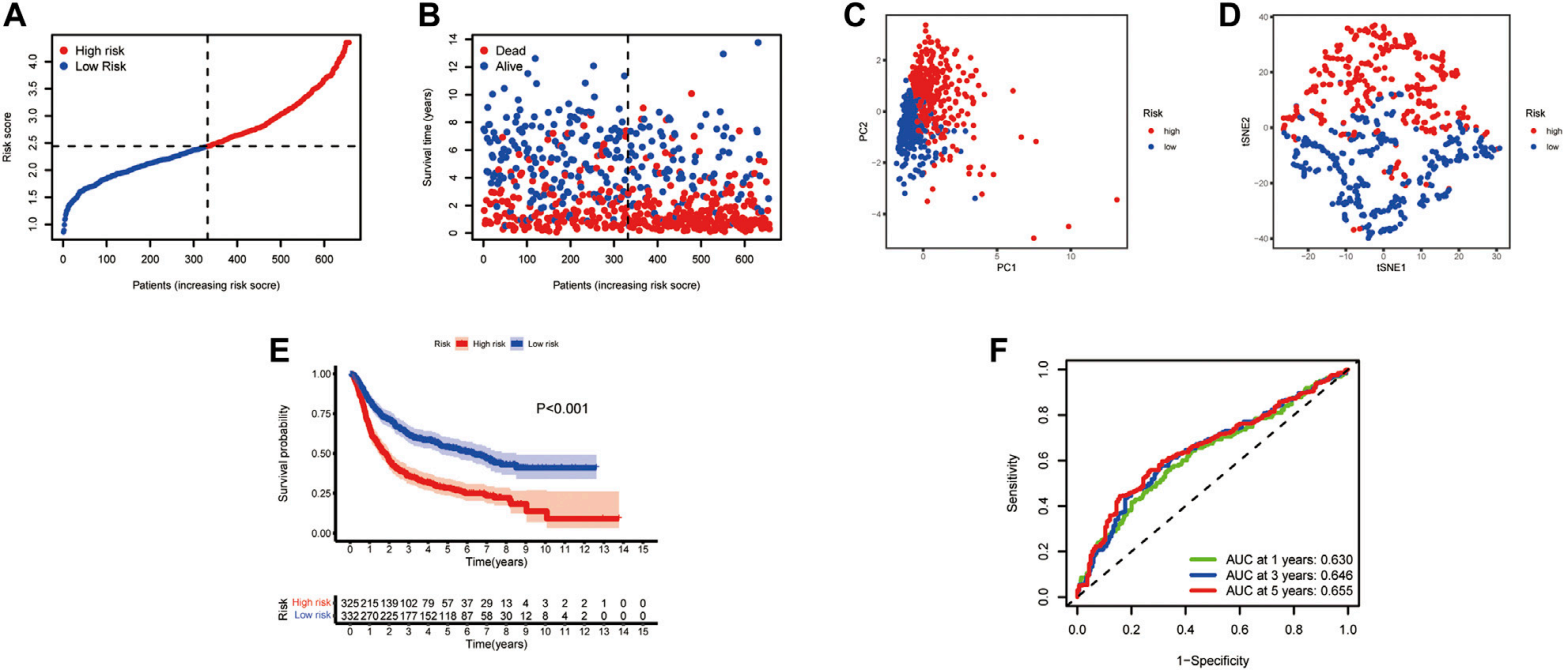
Validation of the risk signature in the CGGA testing set. **(A)** Patient distribution and median value of CR-Score. **(B)** Survival time and status distribution by CR-Score. **(C)** PCA plot of the CGGA cohort. **(D)** t-SNE plot of the dataset. **(E)** Kaplan‐Meier survival analysis of OS for glioma patients. **(F)** Time-dependent ROC curves.

In this study, 693 glioma samples from the CGGA cohort were used as the test set. Before proceeding further, we used the same formula to normalize the RNA sequencing expression data. Patients were divided into high-risk groups (*n* = 325) and low-risk groups (*n* = 332) according to the median cutoff value of the TCGA cohort (Figure 4A). Our analysis indicated that the high-risk group had a worse survival than the low-risk group (Figures 4B–E). The PCA and t-SNE results also showed a satisfactory separation between the two groups (Figures 4C,D). Our model predicted 1-, 3-, and 5-year OS with AUCs were 0.630, 0.646, and 0.655, respectively (Figure 4F). Analysis of the five CXCR-based prognostic signature showed that the CR-Score was still comparatively performing well, suggesting that the CR-Score can accurately predict the clinical outcome of glioma patients.

### Independent Prognostic Value of the CXC Chemokine Receptor Molecules

**FIGURE 5:**
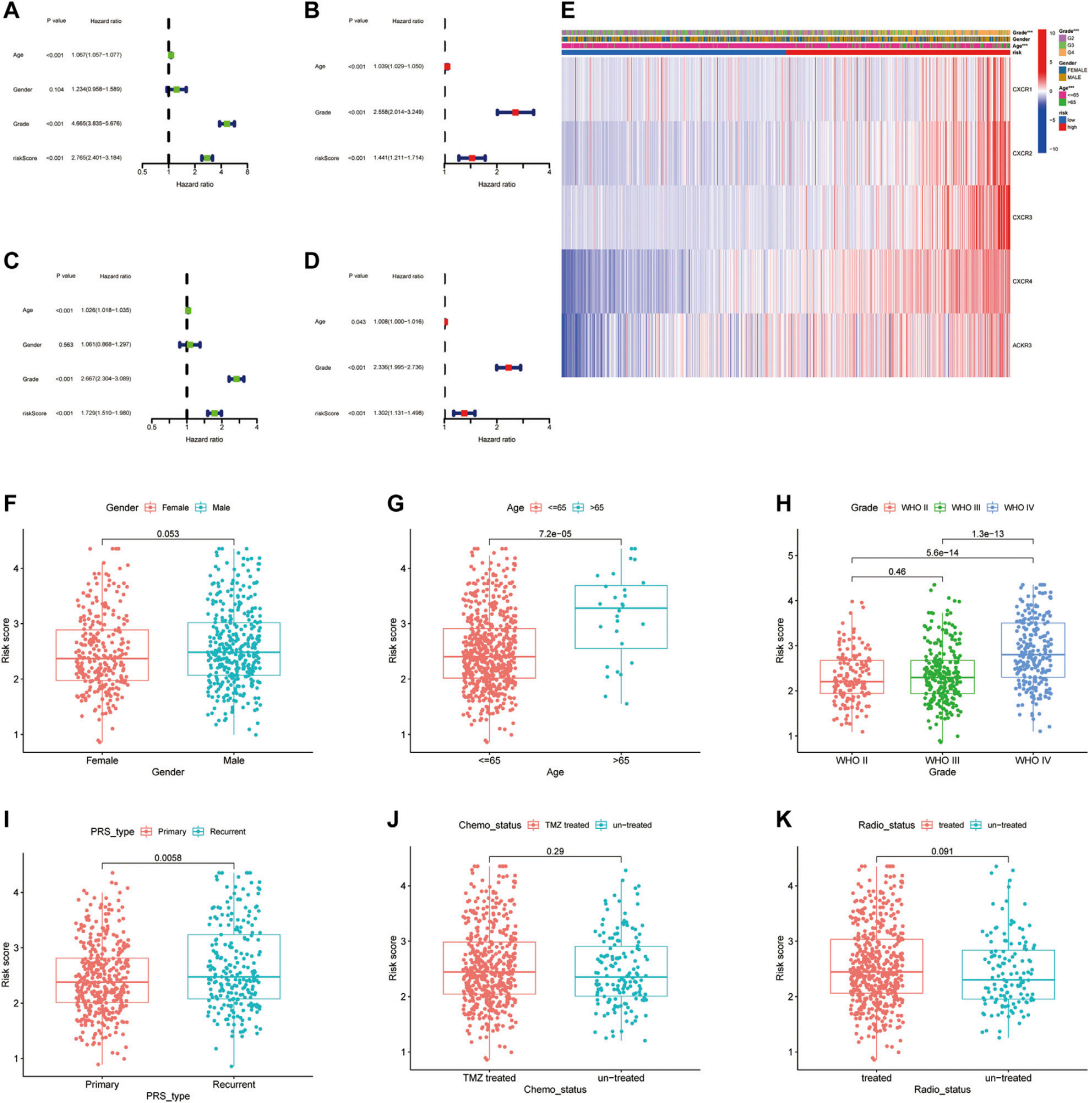
Independent prognostic analysis of CR-Score and clinicopathological parameters. Forrest plot of univariate (**A**, Green) and multivariate (**B**, Red) Cox regression analysis in the TCGA cohort. Univariate **(C)** and multivariate **(D)** Cox regression analysis in the CGGA cohort. **(E)** The relationship between CR-Score and clinicopathologic indicators. **(F–K)** Combination of CR-Score with gender, age, grade, primary/recurrent status, chemotherapy status, radiation therapy.

We then performed a univariate and multivariable Cox proportional hazard analysis to determine whether the CXCR signature model could be used as an independent prognostic indicator for glioma patients. Univariate Cox regression analysis showed that age, grade, and CR-Score were significantly associated with prognosis. The higher the CR-Score, the worse the prognosis (HR: 2.765, 95%CI: 2.401–3.184, *p* < 0.001 Figure 5A; HR: 1.729, 95% CI: 1.510–1.980, *p* < 0.001 Figure 5C). After adjusting for potential confounding factors, the CR-Score also showed significance in multivariate Cox regression (HR: 1.441, 95% CI: 1.211–1.714, *p* < 0.001, Figure 5B; HR: 1.302, 95% CI: 1.131–1.498, *p* < 0.001, Figure 5D). Moreover, we conducted a heatmap to interpret the possible associations between the clinicopathological parameters of the TCGA cohort and five genes (Figure 5E). Wilcoxon signed-rank tests compared differences in CR-Score among different groups for these clinicopathological features, indicating that age, clinical stage, and tumor PRS status were positively associated with CR-Score (Figures 5F–K).

### Functional Enrichment and Immune Infiltrating

**FIGURE 6:**
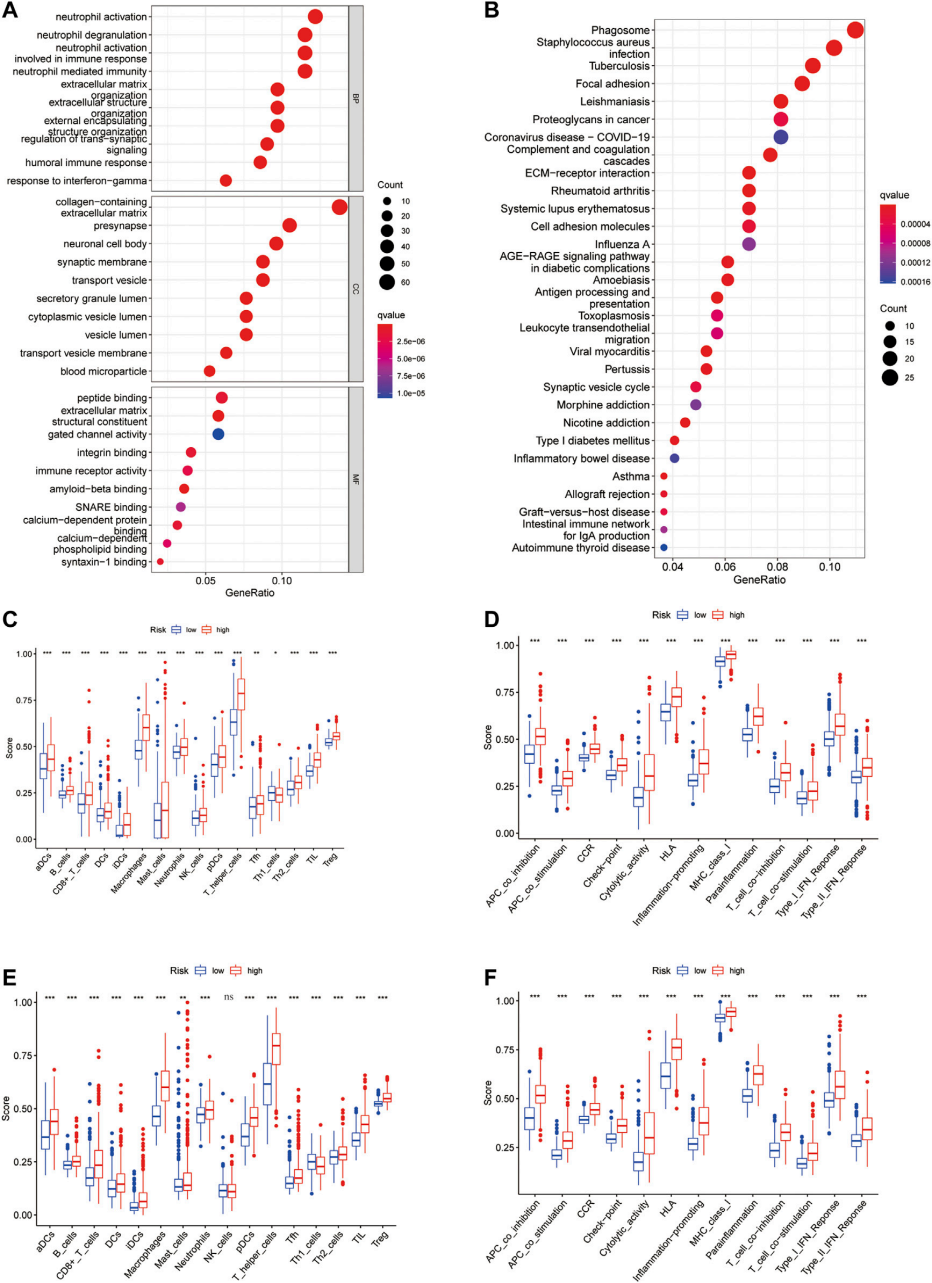
Functional annotation enrichment analysis and comparison of immune cell infiltration between CR-Score subgroups. **(A)** GO functional enrichment analysis. **(B)** KEGG functional enrichment analysis. Bubble size and color correspond to the differentially enriched gene number and *p*-value for the significance of the enrichment. Boxplots of ssGSEA results for 16 immune cells **(C)** and 13 immune-related functions **(D)** in the TCGA cohort. The scores of 16 immune cells **(E)** and 13 immune-related functions **(F)** in the CGGA cohort. Data are shown as the mean ± SD; *t* test, *p < 0.05, **p < 0.01 and ***p < 0.001 compared to the high-risk group in **(C–F)**; ns, no significance.

To elucidate the functions of CXCR-related genes between the two subgroups classified from the risk model, we extracted DEGs in the TCGA cohort with the “limma” R package with | log2FC | ≥ 1 and FDR<0.05. A total of 470 DEGs were identified between the high- and low-risk groups. Among them, 261 genes were upregulated and 209 genes were downregulated (Supplementary Table S4). Based on these DEGs, analyses of GO functional annotation and KEGG pathway enrichment were performed. The DEGs were mainly enriched in immune biology processes such as neutrophil activation/degranulation, neutrophil-mediated immunity, and regulation of trans−synaptic signaling in GO analysis (Figure 6A). In the KEGG pathway enrichment analyses, we identified DEGs involved in the Phagosome, Focal adhesion, Proteoglycans in cancer, ECM−receptor interaction, Coronavirus disease—COVID-19, ECM−receptor interaction, and Cell adhesion molecules (Figure 6B). To find out the relationship between CR-Score and immune infiltrations, the scores of 16 immune cells and 13 immune-related functions were assessed using the ssGSEA method in the GSVA package. Remarkably, the scores of the immune cell types (including aDCs, B_cells, CD8+_T_cells, Macrophages, Neutrophils, T-helper-cells, TIL, and Treg) were considerably different between these two risk groups (Figures 6C–E). In addition, all 13 immune-related signaling pathways also differed between the low- and high-risk groups (Figures 6D–F).

### Variation in the Infiltration Profiles of Tumor Microenvironment cells

**FIGURE 7:**
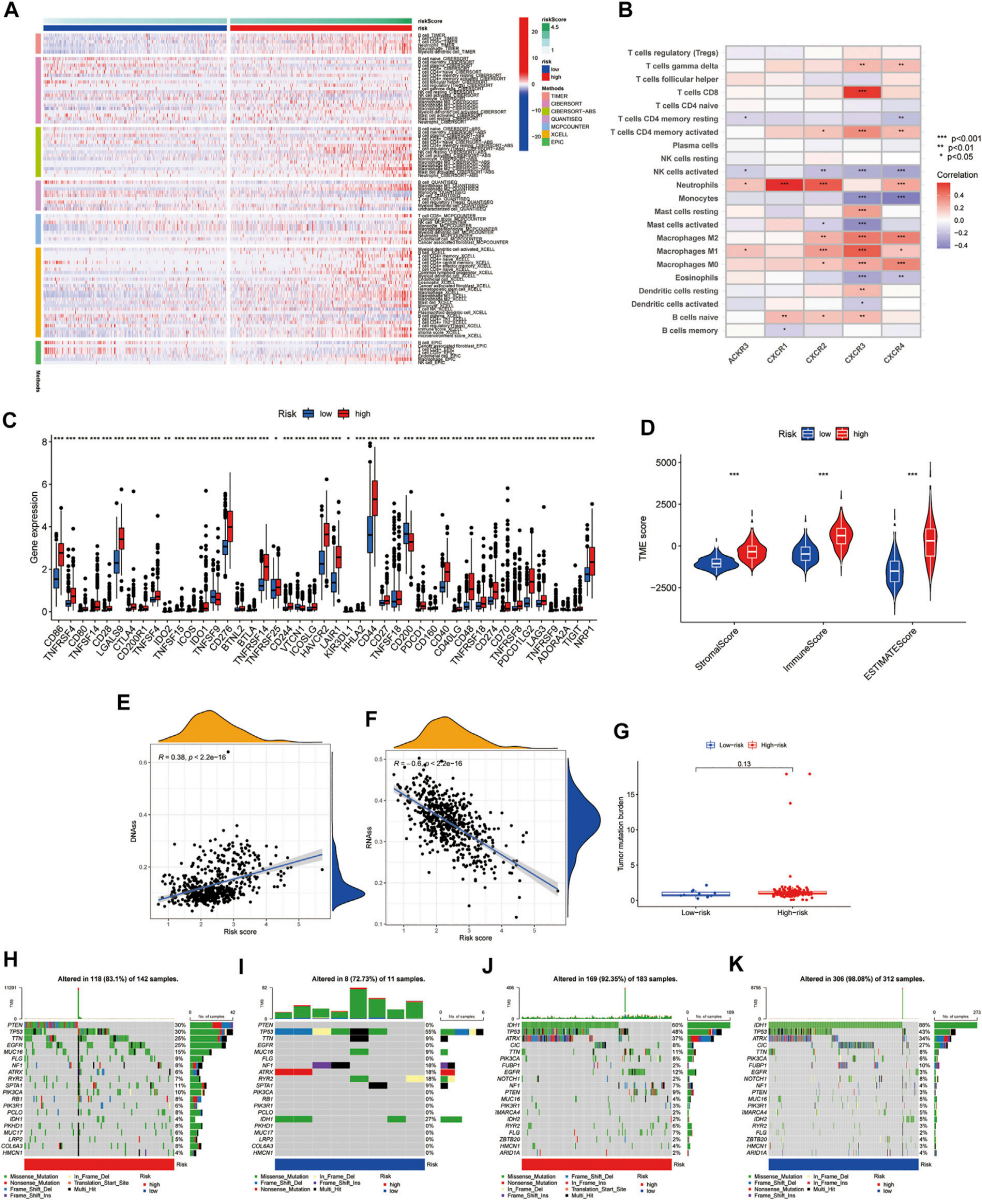
Correlation of CR-Score and with TME, CSC index, immune checkpoints, and mutation. **(A)** The thermogram indicates the frequency of TME infiltrating cells among high- and low-risk group. **(B)** Relationships between the infiltrating levels of 22 immune cell types and the differentially expressed genes. **(C)** The expression of immune checkpoint genes. **(D)** Associations between the CR-Score and TME score. Relationships between CR-Score and CSC index. **(E)** DNAss. **(F)** RNAss. **(G)** Correlations between CR-Score and TMB. **(H–K)** Waterfall plot of somatic mutation profiles established with high and low CR-Scores in each sample. Data are shown as the mean ± SD; t test, *p < 0.05, **p < 0.01 and ***p < 0.001 compared to the high-risk group in **(B–D)**.

We use Timer, Cibersort, Quantiseq, Mcpcounter, Xcell, and Epic to estimate the abundances of immune cells infiltrating in glioma samples using mRNA-Sequencing data. Patients with high CR-Score accumulated more tumor-infiltrating immune cells such as T cell CD8^+^, neutrophil, macrophage, and myeloid dendritic cells (Figure 7A).

The relationship between the expression of five genes of the proposed model and infiltrating immune cells was also investigated. We observed that Neutrophils, NK cells activated, and Macrophages M2 were significantly related to CXCR genes (Figure 7B). In addition, we found that immune checkpoint-related genes, including PD-1, PD-L1, and CTLA-4, were overexpressed in high-risk patients compared to low-risk patients (Figure 7C). Tumor microenvironment analysis was also performed, the higher the StromaScore or ImmuneScore, the higher the relative content of stroma or immune components in the immune microenvironment, and the ESTIMATEScore represented the accumulation of stroma or immune cells. We found that subtype C2 had higher TME scores than subtype C1 (Figure 7D). Figures 7E,F showed the linear correlation between the CSC index and the CR-Score in glioma. We found that the CR-Score correlated positively with the DNAss index (R = 0.38, *p* < 0.001), while the RNAss index correlated negatively with the CR-Score (R = −0.6, *p* < 0.001), indicating that glioma cells with a higher CR-Score had a higher degree of differentiation and more stem cell characteristics (Figures 7E,F).

Accumulating evidence suggested that patients with high TMB status may benefit from preventive immunotherapy due to a higher proportion of tumor-specific neoantigens. However, a pooled analysis of TMB showed no significant difference between the two risk groups (Figure 7G). GBM patients with high CR-Scores had significantly higher frequencies of PTEN, TTN, and EGFR mutations compared with patients with low CR-Scores. However, the mutation levels of TP53 and NF1 were the exact opposite (Figures 7H,I). The somatic mutation features of LGG established with high and low CR-Score were shown in Figures 7J,K.

### Establishment of a Nomogram Model for the Prognosis of Glioma.

**FIGURE 8:**
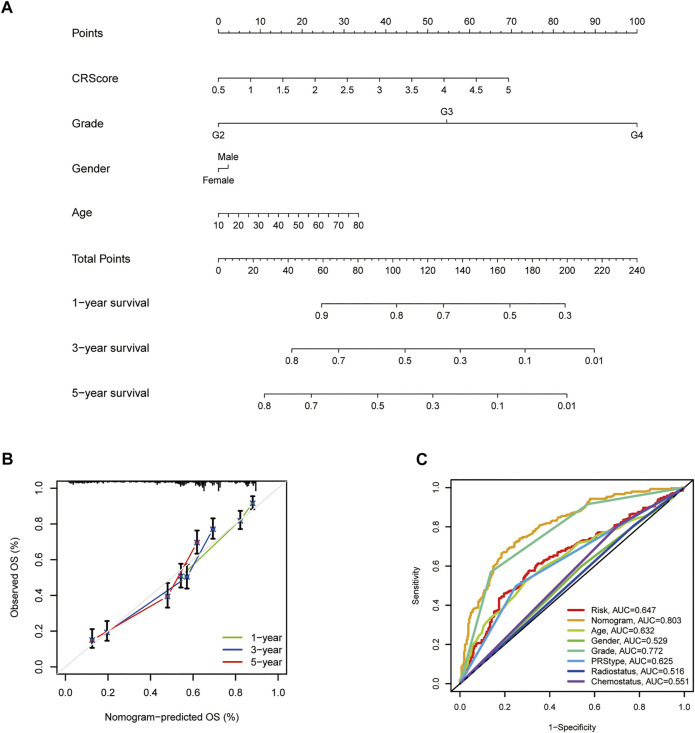
Establishment and application of the scoring model in the external validation cohort. **(A)** Nomogram for the prediction of OS at 1, 3, and 5 years. **(B)** The calibration curves for external validation of the nomogram. **(C)** The clinical ROC curves of the CXCR-related nomogram at 3-year OS.

Given the inconvenience of CR-Score in the clinical application, a prognostic nomogram model was established to predict the probability for glioma patients (Figure 8A). The predictors included CR-Score, tumor grade, patient age, and gender. The calibration plot of the nomogram showed that the 1-year, 3-years, and 5-year OS rates can be better predicted in the CGGA cohort (Figure 8B). Next, clinical ROC curves were performed to assess the sensitivity and specificity of the nomogram. The AUC of this nomogram at 3- year survival reached 0.803, indicating the potential clinical values of the nomogram model (Figure 8C).

## Discussion

Increasing evidence suggests the crucial role of CXCR in anti-tumor immunity, however, the underlying molecular mechanism of glioma is still not fully understood. In this research, we explored the transcriptional and genetic heterogeneity of seven CXCR molecules in both tumor tissues and normal tissues and found that the regulation of genome variation may not be related to the degree of CXCR expression. Then we identified two distinct molecular subtypes of CXCR in glioma. Significant differences in the immune cell infiltration level and clinical characteristics among different clusters were observed. The success of ICI depends on prior recruitment of the TILs, particularly the existence of CD8 + T cells, in the TME. It is generally believed that the extent of PD-L1 and PD-1 expression correlates with better immunosuppressive therapy (Topalian et al., 2012). In our research, the group with a high CR-Score had more checkpoint molecular expression and ESTIMATE score. The results show that there is a significant correlation between CXCR and tumor immunity in glioma. Consequently, we confirmed that the combination of CXCR inducers and ICI has great potential for the development of new combined therapeutic strategies. In addition, compared with PD-L1 protein expression detected by immunohistochemical, a high TMB is more significantly associated with better response to PD-1/PD-L1 blockades (Luchini et al., 2019). However, we did not detect any significant differences in the TMB between the two risk groups.

We focused on five CXCR genes, CXCR1/2/3/4/7, which have a significant impact on the overall survival rate of glioma. There is a high degree of homology between CXCR1 and CXCR2, studies from Lee et al. showed that knockdown of CXCR1 or CXCR2 was effective in inhibiting neutrophilic infiltration and tumor growth *in vitro* and *in vivo* (Lee et al., 2012). CXCR3 is a CXC chemokine receptor dominated by IFN-γ, which interacts with CXCL9, CXCL10, and CXCL11 to regulate tumor progression and cytokine secretion (Singh et al., 2013). This is consistent with the results of our current study. Recently, Saahene et al. showed that CXCL4 interaction with CXCR3b might be associated with poor prognosis in breast cancer (Saahene et al., 2019). Among the CXCRs, CXCR4 is the most studied in gliomas. Over-expression of CXCR4 has been identified as a promising prognostic biomarker for gastrointestinal and acute myeloid leukemia, among many other tumor types (Du et al., 2019; Jiang et al., 2019). High expression of CXCR4 in glioma was modulated through Akt/mTOR signaling by Notch1, which promotes the migration of glioma-originating cells (Zheng et al., 2018). In the nervous system, the CXCR7/CXCL12 signaling pathway regulates the differentiation and growth of astrocytes, Schwann cells as well as glioma cells (Odemis et al., 2010; Ödemis et al., 2012). Previous studies have indicated that CXCR7 antagonists could suppress tumor activation in animal models (Burns et al., 2006). So far, little was known about the role of CXCR5 in glioma. Studies carried out by Zheng et al. reported that the CXCR5-CXCL13 axis promotes the growth of colorectal cancer and clear cell renal cell carcinoma (ccRCC) *via* activating PI3K/Akt/mTOR signaling (Zhu et al., 2015; Zheng et al., 2018). In this study, the results indicated that the mRNA expression of CXCR5 in glioma was higher than that in normal samples. However, CXCR5 was not correlated to the prognosis of glioma. Similarly, CXCR6 is highly expressed in multiple tumor types, and the CXCR6-CXCL16 axis is mainly related to NF-κB and PI3K/Akt signaling pathways. Lepore et al. reported that CXCR6 knockout greatly prolonged survival in mice (Lepore et al., 2018). Unfortunately, the underlying mechanism of CXCR6 is still unknown. In the present study, we found that the expression of CXCR6 between glioma and normal brain tissue did not show any significant difference.

Considering the intratumoral heterogeneity of CXCR phenotypes in individuals with glioma, we established a clinical risk scoring system, CR-Score, to evaluate the value of CXCR molecules in glioma patients. There is a significant positive correlation between the CR-Score and immune cell infiltration level in glioma. In clinical practice, CR-Score can be used to selectively evaluate the immune cell infiltration of TME and the corresponding expression pattern of CXCR-related molecules in glioma patients, confirm the tumor immunophenotype, predict the prognosis of individual patients, and inform the medication properly. In summary, our research explored genetic and transcriptional levels of CXCR-related molecules in glioma and showed that CXCR molecules play a significant role in the remodeling of the tumor microenvironment. These results have strengthened our understanding of the tumor immune microenvironment, improved the response of patients to immunotherapy, identified two distinct tumor immunophenotypes, and promoted precise cancer immunotherapy in the future.

We recognize that our study has some limitations. This is a two-center retrospective study. Although we have corrected the batch effect to a large extent and conducted independent cohort experiments, the current research still has some impact. Further in-depth experimental studies are needed. Our results indicate that CXCR1/2/3/4/7 may play a significant role in glioma, but there is still a lack of investigation on the exact molecular mechanism of oncogenes involved. We are currently collecting samples in a multicenter clinical cohort for further verification and analysis.

## Data Availability

The datasets presented in this study can be found in online repositories. The names of the repository/repositories and accession number(s) can be found in the article/Supplementary Material.

## Abbreviations

GBM, Glioblastoma; LGG, Low-grade glioma; TCGA, The Cancer Genome Atlas; CSC, Cancer stem cell; CNV, Copy number variation; TME, Tumor microenvironment; TILs, Tumor-infiltrating lymphocytes; ICIs, Immune Checkpoint Inhibitors; GO, Gene Ontology; KEGG, Kyoto Encyclopedia of Genes and Genomes; OS, Overall survival; TIICs, Tumor-infiltrating immune cells; ROC, Receiver Operating Characteristic; AUC, Area Under the Curve.
