# Supplementary material for: Prognostic Value and Therapeutic Perspectives of CXCR Members in the Glioma Microenvironment
Source: Front Genet. 2022 Apr 27;13:787141. doi: 10.3389/fgene.2022.787141 (PMC9091590; doi:10.3389/fgene.2022.787141)
Supplement: Supplementary file 2 [file DataSheet1.DOCX]

**Supplementary Figures**


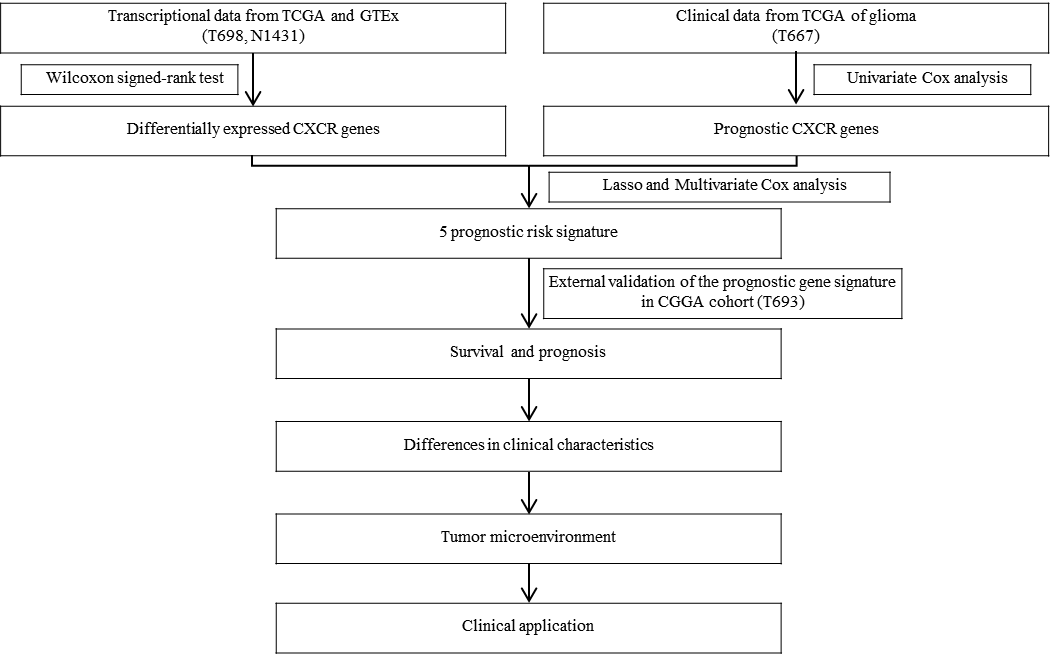


**Figure S1** Workflow chart of this study.


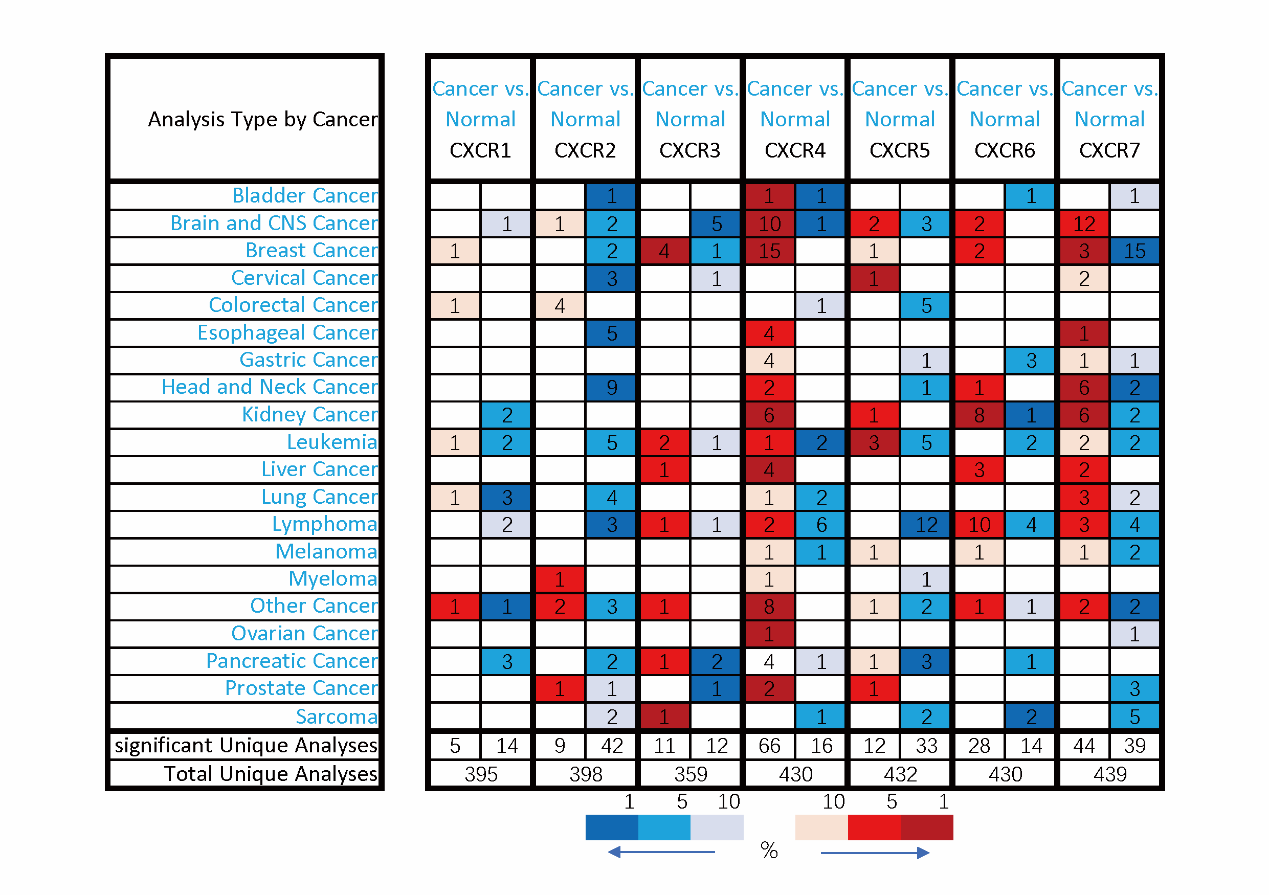


**FIGURE S2** Figure 1. The mRNA expression of CXCRs in different types of cancers (Oncomine) Red shows up-regulation and blue reveals down-regulation, The cut-off point of p-value was set at 0.01 and the threshold of the fold change was defined as 1.5, gene rank: 10%, data type: mRNA, respectively.
